# Sexual dimorphism of *AMBRA1*-related autistic features in human and mouse

**DOI:** 10.1038/tp.2017.213

**Published:** 2017-10-10

**Authors:** M Mitjans, M Begemann, A Ju, E Dere, L Wüstefeld, S Hofer, I Hassouna, J Balkenhol, B Oliveira, S van der Auwera, R Tammer, K Hammerschmidt, H Völzke, G Homuth, F Cecconi, K Chowdhury, H Grabe, J Frahm, S Boretius, T Dandekar, H Ehrenreich

**Affiliations:** 1 Department of Clinical Neuroscience, Max Planck Institute of Experimental Medicine, Göttingen, Germany; 2 DFG Research Center for Nanoscale Microscopy and Molecular Physiology of the Brain (CNMPB), Göttingen, Germany; 3 Department of Psychiatry and Psychotherapy, UMG, Georg-August-University, Göttingen, Germany; 4 Biomedizinische NMR Forschungs GmbH, Max Planck Institute for Biophysical Chemistry, Göttingen, Germany; 5 Department of Bioinformatics, Biocenter, University of Würzburg, Würzburg, Germany; 6 Department of Psychiatry and Psychotherapy, University Medicine, and German Center for Neurodegenerative Diseases (DZNE) Greifswald, Greifswald, Germany; 7 Cognitive Ethology Laboratory, German Primate Center, Göttingen, Germany; 8 Institute for Community Medicine, University Medicine Greifswald, Greifswald, Germany; 9 Interfaculty Institute for Genetics and Functional Genomics, University of Greifswald, Greifswald, Germany; 10 IRCCS Fondazione Santa Lucia and Department of Biology, University of Rome Tor Vergata, Rome, Italy; 11 Unit of Cell Stress and Survival, Danish Cancer Society Research Center, Copenhagen, Denmark; 12 Department of Molecular Cell Biology, Max Planck Institute of Biophysical Chemistry, Göttingen, Germany; 13 Department of Functional Imaging, German Primate Center, Leibniz Institute of Primate Research, Göttingen, Germany

## Abstract

*Ambra1* is linked to autophagy and neurodevelopment. Heterozygous *Ambra1* deficiency induces autism-like behavior in a sexually dimorphic manner. Extraordinarily, autistic features are seen in female mice only, combined with stronger Ambra1 protein reduction in brain compared to males. However, significance of *AMBRA1* for autistic phenotypes in humans and, apart from behavior, for other autism-typical features, namely early brain enlargement or increased seizure propensity, has remained unexplored. Here we show in two independent human samples that a single normal *AMBRA1* genotype, the intronic SNP rs3802890-AA, is associated with autistic features in women, who also display lower *AMBRA1* mRNA expression in peripheral blood mononuclear cells relative to female GG carriers. Located within a non-coding RNA, likely relevant for mRNA and protein interaction, rs3802890 (A versus G allele) may affect its stability through modification of folding, as predicted by *in silico* analysis. Searching for further autism-relevant characteristics in *Ambra1*^+/−^ mice, we observe reduced interest of female but not male mutants regarding pheromone signals of the respective other gender in the social intellicage set-up. Moreover, altered pentylentetrazol-induced seizure propensity, an *in vivo* readout of neuronal excitation–inhibition dysbalance, becomes obvious exclusively in female mutants. Magnetic resonance imaging reveals mild prepubertal brain enlargement in both genders, uncoupling enhanced brain dimensions from the primarily female expression of all other autistic phenotypes investigated here. These data support a role of *AMBRA1/Ambra1* partial loss-of-function genotypes for female autistic traits. Moreover, they suggest *Ambra1* heterozygous mice as a novel multifaceted and construct-valid genetic mouse model for female autism.

## Introduction

Autism spectrum disorders (ASD) are extremely heterogeneous neurodevelopmental conditions, affecting ~1% of the population. Typical, shared symptoms range from social communication and interaction deficits, including decreased attraction by and compromised reading of social signals, restricted interests, repetitive behaviors or pronounced routines, to reduced cognitive flexibility.^1, 2, 3, 4^ Early brain enlargement^5, 6, 7, 8^ and predisposition to epileptic seizures^9, 10^ are among the reported non-behavioral characteristics found in this disorder category. Causes likely converge at the synapse, as indicated by mutations of synaptic genes or by mutations causing quantitative alterations in synaptic protein expression, half-life or degradation, and are reflected by a virtually autism-pathognomonic neuronal excitation–inhibition dysbalance.^4, 11, 12, 13, 14, 15^ Neuroligin-4 mutations, for instance, belong to the most common causes of monogenetic heritable autism.^16^ Construct-valid and face-valid mouse models of autism, building on monogenetic grounds, have helped in approaching the underlying common biology.^17, 18^

The estimated heritability of autism approximates 90%. We note, however, that monogenetic forms including copy number variations altogether account for <20%, leaving ~80% of cases unexplained, which also enter the final common pathway of disease expression.^1, 2, 3^ Importantly, normal genetic variants, mainly single nucleotide polymorphisms (SNPs), likely contribute to the manifestation of autistic phenotypes. This is indicated by the results of genome-wide association (GWAS) and respective polygenic-risk studies on autism,^2, 19, 20^ but even more so by phenotype-based genetic association studies (PGAS), reporting an accumulation of ‘unfortunate’ normal variants, so-called pro-autistic genotypes, to be associated with increasing severity of autistic traits.^21, 22, 23^ In fact, phenotypical continua of autistic features from health to disease suggest underlying mechanisms of quantitative rather than qualitative nature.^21, 24^ Genetic modifiers, like protective genes and environmental (co-)factors, mainly those acting during uterine and early postnatal development may also modulate autism severity.^25, 26^

ASD has an overall gender distribution of ~4:1 males/females.^1, 2, 27^ Remarkably, little research has focused on the reasons for this disparity. Better understanding of this difference, however, could lead to major advancements in the prevention or treatment of ASD in both genders.^28^ We previously reported that an *Ambra1* (activating molecule in Beclin1-regulated autophagy) partial loss-of-function genotype is associated with the autism-like behavior in female mice. The restriction to the female gender of autism generation by a defined genetic trait has thus far remained unique.^29^ Ambra1 is a positive regulator of a principal player in autophagosome formation, Beclin1. Importantly, autophagy has already been linked to autism in recent work.^30, 31^ Moreover, Ambra1 is involved in other developmentally relevant processes in the nervous system and in neuronal function.^32, 33^ Although homozygosity of the *Ambra1* null mutation causes embryonic lethality, heterozygous mice with reduced Ambra1 expression appear completely normal at first view.^33^ Only upon comprehensive behavioral characterization, a striking autism-like phenotype of *Ambra1*^+/−^ females emerges. This trait is quantifiable by the autism severity composite score, which even allows a behavior-based genotype predictability of >90%.^29, 34^ As first mechanistic hint explaining the prominent gender difference, stronger reduction of Ambra1 protein in the cortex of *Ambra1*^+/−^ females was found.^29^

Until now, no association of *AMBRA1* genotypes with autistic features has been described in humans, therefore still questioning the construct-validity of our mouse model.^29^ However, a recent GWAS on schizophrenia identified a genetic risk variation on chromosome 11 (11p11.2) in a region containing *AMBRA1.*^35^ Schizophrenia and ASD show considerable syndromic overlap, including deficits in social cognition and communication,^24, 36^ and at least a subgroup of schizophrenia is also regarded as a disorder of the synapse.^37^

The present study has therefore been designed to explore whether any autism-relevant phenotype association with normal *AMBRA1* genotypes would emerge in humans, thereby supporting the construct-validity of our *Ambra1*^+/−^ mouse model. In addition, we aimed at defining potential further characteristics of ASD in *Ambra1*^+/−^ mice, namely (1) decreased interest in social odors, as highly relevant social signals in mice, (2) increased epileptic predisposition as *in vivo* readout of neuronal excitation–inhibition dysbalance and (3) early brain enlargement, as recognized in human autism. Indeed, we show here that also in humans, an *AMBRA1* genotype, the intronic SNP rs3802890-AA, located in a long non-coding (lnc) RNA, is linked to autistic features and characterized by partial loss-of-function in females. Moreover, we demonstrate in *Ambra1*^+/−^ mice prepubertal brain enlargement. Only in female mutants, we see loss of interest in sex pheromones and altered seizure propensity.

## Materials and methods

In all experiments, the experimenters were unaware of genotypes (‘fully blinded’).

### Human studies

#### Discovery: schizophrenia subjects and healthy controls (GRAS)

The Göttingen Research Association for Schizophrenia (GRAS) data collection consists of >1200 deep-phenotyped patients, diagnosed with schizophrenia or schizoaffective disorder (DSMIV-TR^38^), recruited across Germany since 2005.^39, 40^ Diagnosis is based on a comprehensive examination, lasting for at least 4 h (the examination often took much longer, with breaks in between, dependent on the patient’s condition). It is guided by the GRAS manual, which contains standardized interviews, psychopathology and neuropsychology testing. Moreover, careful study of all the medical discharge letters and charts of every single individual aids in assessing longitudinal aspects of the diagnosis as well.^39, 40^ GRAS, complying with Helsinki Declaration, was approved by Ethics Committees of Georg-August-University, Göttingen, Germany, and participating centers. All study participants (European-Caucasian 95.6% other 1.8% unknown 2.6%) and, if applicable, legal representatives gave written informed consent. Of the 1105 successfully genotyped patients, 66.7% were male (*N*=737), 33.3% female (*N*=368), aged 39.46±12.58 years (range: 17–79). For genetic case–control analysis, healthy GRAS blood donors were employed,^39^ in total *N*=1258 (European-Caucasian 97.8% other 2% unknown 0.2%), 61.6% male (*N*=775), 38.4% female (*N*=483), aged 37.45±13.21 (range: 18–69) years. Voluntary blood donors widely fulfill health criteria according to the national guidelines for blood donation, ensured by a broad pre-donation screening process containing standardized questionnaires, interviews, hemoglobin, blood pressure, pulse and body temperature determinations.^39^

#### Replication: population-based cohort (SHIP-O)

The general population sample comprises *N*=2359 homozygous subjects, mean age 49.8±16 (range=20–81) years, *N*=1144 males, *N*=1215 females, from baseline examinations of Study of Health in Pomerania (SHIP), approved by the Ethics Committee, University Greifswald, and conducted in North-East Germany.^41^

#### Phenotyping

**Figure 1:**
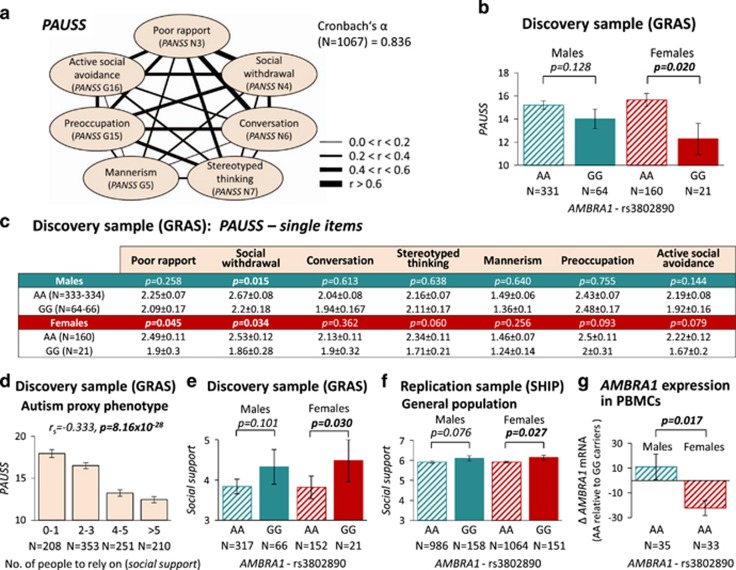
Human *AMBRA1-*rs3802890 G/A: association with autistic features. (**a**) Quantification of autistic symptoms using *PAUSS* (PANSS Autism Severity Score.^24^ Note the high intercorrelation of *PAUSS* items and the high internal consistency of the scale (Spearman rank correlation coefficients; Cronbach’s *α*). (**b**) PGAS using *AMBRA1*-rs3802890 and *PAUSS* score: female AA subjects display a higher *PAUSS* score than GG subjects in the discovery sample; mean±s.e.m.; two-tailed Mann–Whitney *U*-test (data-corrected by linear regression analysis for age). (**c**) Trends of positive association between rs3802890-AA genotype and sub-items of *PAUSS,* more pronounced in females; mean±s.e.m.; two-tailed Mann–Whitney *U*-test. (**d**) The highly significant correlation of *PAUSS* and social support underlines the validity of social support as an autism proxy phenotype; mean±s.e.m. (**e**) Genotype effect of *AMBRA1*-rs3802890 on degree of social support in the discovery sample, again significant in females; mean±s.e.m.; Mann–Whitney *U-*test. (**f**) Replication of the genotype and gender effect of *AMBRA1*-rs3802890 using social support as proxy in the general population; linear regression analyses (bootstrap; data-corrected for age); mean±s.e.m. (**g**) Relative *AMBRA1* mRNA expression in peripheral blood mononuclear cells (PBMC) is reduced in female AA (risk SNP) carriers: shown is the *AMBRA1* mRNA expression in AA carriers, given as mean Δ-value compared to GG carriers (GG males *N*=33; GG females, *N*=14). Values of individual AA males (*N*=35) and AA females (*N*=33) are expressed in %GG (mean) of the respective gender. The Δ-value is calculated as: Δ=%GG–100% mean±s.e.m.; two-tailed Mann–Whitney *U-*test.

For quantification of autistic phenotypes, we used the Positive and Negative Syndrome Scale (PANSS)^42^-based autism severity score (PAUSS)^24^ with slight modifications (Figure 1a), available for 1067 patients. For the replication sample, the Instrumental Support Index (ISI) was taken as proxy, indicating quality of instrumental and emotional support,^43^ expected to be low in autistic individuals.^23^ It was cross-validated with PAUSS in GRAS subjects, with social support operationalized as self-reported number of individuals a person can rely on in case of emergency.^23^ For both measures of social support (intercorrelation 0.77), higher score values (*z*-transformed; range: 1.5–9) represent higher social support, that is, lower autistic features.

#### Genotyping

GRAS subjects were genotyped using semi-custom Axiom MyDesign Genotyping Array (Affymetrix, Santa Clara, CA, USA), based on a CEU (Caucasian residents of European ancestry from UT, USA) marker backbone, including 518 722 SNPs, plus custom marker-set of 102 537 SNPs. Genotyping was performed by Affymetrix on a GeneTitan platform with high quality (SNP call rate >97%, Fisher’s linear-discriminant, heterozygous cluster-strength offset, homozygote-ratio offset).^23, 44, 45^ Markers were selected according to our SOP for PGAS^23^ using following selection criteria: (1) SNPs in Hardy–Weinberg equilibrium; (2) SNPs with minor allele frequency (MAF⩾0.2) allowing for statistical analyses; (3) SNPs not in high linkage disequilibrium (LD) with other selected SNPs (*r*^2^<0.8). Based hereon, only rs3802890-A/G remained for analysis.

SHIP-0 subjects were genotyped using Affymetrix Genome-Wide SNP Array-6.0 (genotyping efficiency 98.6%). Imputation of genotypes was performed with software IMPUTE v0.5.0^46^ against 1000-Genomes (phase1v3) reference-panel using 869 224 genotyped SNPs.^41^ Rs3802890 was imputed with IQ=1.

#### *In silico* analyses

Genome sequences were established according to latest available releases (human-genome vs32–2015; mouse-genome 2016). Iterative sensitive sequence comparisons were conducted^47^ and evaluated including detailed genome and transcriptome mapping. Expression of the rs3802890-containing RNA region was derived from latest largest collection of ESTs available at NIH.^48^ LncRNA matches were also established according to latest human lncRNA release at NCBI. RNA folding used RNAfold.^49^ For demonstrating rs3802890-A/G differences, thermodynamic ensemble structures drawing encoded base-pair probabilities were used. Protein binding regions were calculated using RNAanalyzer^50^ and CatRapid,^51^ coding potential was calculated using Genscan.^52^

#### AMBRA1 and EST TCAAP2E6309 mRNA expression

Peripheral blood mononuclear cells (PBMC) were isolated from morning blood, obtained via phlebotomy into CPDA-vials (Citrate-Phosphate-Dextrose-Adenine, Sarstedt, Germany), applying standard Ficoll-Paque-Plus isolation procedure (GE-Healthcare, Munich, Germany). Total RNA extraction was done using miRNeasy Mini-kit (Qiagen, Hilden, Germany). For reverse transcription*,* 1 μg of cDNA was applied using a mixture of oligo(dT)/hexamers, dNTPs, DTT and 200U SuperscriptIII (Life Technologies, Darmstadt, Germany). *AMBRA1* RNA expression was measured using quantitative real-time PCR. The cDNA was diluted 1:25 in 10 μl reaction-mix, containing 5 μl of SYBR-green (Life Technologies) and 1pmol/primer:

*AMBRA1*-Fw: 5′-GACCACCCAATTTACCCAGA-3′

*AMBRA1*-Rv: 5′-GATCATCCTCTGGGCGTAGTA-3′

*GAPDH*-Fw: 5′-CTGACTTCAACAGCGACACC-3′

*GAPDH*-Rv: 5′-TGCTGTAGCCAAATTCGTTGT-3′

Technical triplicates were run on LightCycler480 (Roche-Diagnostics, Mannheim, Germany). Relative *AMBRA1* expression was calculated using the threshold-cycle method (LightCycler480 Software1.5.0SP3-Roche) and normalization to GAPDH. EST TCAAP2E6309 RNA expression was measured using traditional PCR. Extracted RNA, cDNA synthesized with oligo-dT primers with/without hexamers, or genomic DNA were used as template with the following primers:

EST TCAAP2E6309-Fw: 5′-GGCAGAGCAGAATGGATAGACA-3′

EST TCAAP2E6309-Rv: 5′-AACGCCTGTTATCTGGGATCA-3′

### Mouse studies

Investigations were carried out in agreement with guidelines for welfare of experimental animals, issued by the Federal Government of Germany and Max Planck Society, approved by local animal care and use committee (Niedersächsisches Landesamt für Verbraucherschutz und Lebensmittelsicherheit, Oldenburg, Germany).

#### Mouse line and housing

*Ambra1* mutation in mice is caused by a truncated, non-functional Ambra1 protein via insertion of a gene-trapping vector into the murine *Ambra1* gene.^33^ *Ambra1* wild-type (WT, *Ambra1*^+/+^) and heterozygous (*Ambra1*^+/−^) littermates of both genders with >99% C57BL/6 N background were used (male *Ambra1*^+/−^ × female WT-C57BL/6N). Genotyping was performed as described.^29^ Males and females were kept in separate ventilated cabinets (Scantainers; Scanbur Karlslunde, Denmark), group-housed, with woodchip bedding and nesting material, 12 h-light-dark cycle, 20–22 °C, food/water *ad libitum*.

#### Social intellicage paradigm

For pheromone-based social preference test, *Ambra1*^+/+^ and *Ambra1*^+/−^ mice were separately group-housed in large type4 cages after weaning until age 8 weeks. After transponder implantation, they were put into intellicages (IntelliCage; TSE-Systems, Bad Homburg, Germany), placed inside standard laboratory rodent cages (height 20.5 cm, length 55 cm, width 38.5 cm; Techniplast-Model-2000, Germany) with floor covered by sawdust bedding.^53^ Each intellicage contains four housing shelters beneath the food hopper. Left and right of the intellicage, two social boxes are connected via plastic tubes, each equipped with two ring RFID-antennas to track individual mice. IntelliCage software records time spent in social boxes. Experiments are performed during the light phase. After habituation for 1 h to social boxes containing fresh bedding, mice undergo the pheromone-based social preference test: for 1 h they can freely choose between a social box with used bedding of C3H mice of opposite gender and another box with only fresh bedding. WT mice typically prefer used bedding containing pheromones.

#### Magnetic resonance imaging for morphometry

Mice were anesthetized (5% isoflurane), intubated and kept at 1.75% isoflurane/5% oxygen by active ventilation with constant respiratory frequency (85 breaths per minute; Animal-Respirator-Advanced, TSE-Systems). Magnetic resonance imaging (MRI) was performed at 7 and 9.4T (Bruker Biospin MRI, Ettlingen, Germany). Radiofrequency excitation and signal reception were accomplished with use of a birdcage resonator (inner diameter, 72 mm) and a four-channel phased-array surface-coil, respectively. T2-weighted MRI data were acquired with three-dimensional fast spin-echo MRI sequence (repetition-time TR=3.5 s, effective echo-time TE_eff_=55 ms, 12 differently phase-encoded echoes, 56 min measuring time) at isotropic spatial resolution of 100 μm. From these datasets, polygonal surface models of selected brain structures were generated by importing DICOM images into AMIRA (Visage-Imaging, Berlin, Germany). Structures of interest (whole brain, hippocampus, cerebellum, olfactory bulb, ventricles) were manually and semi-automatically labeled with segmentation editor on three-dimensional label fields (80 horizontal, 192 coronal, 144 sagittal slices).

#### Pentylentetrazol-induced seizures

Mouse groups were tested during the light phase at postnatal day 23 or at 13 months. Seizures were induced by single intraperitoneal injection of pentylentetrazol (PTZ) (50 mg kg^−1^; Sigma-Aldrich, Taufkirchen, Germany). After injection, mice are observed for 30 min in their home cage.^54^ Response to PTZ injection is quantified: (1) hypoactivity: decrease in mobility until rest in crouched or prone position, abdomen at bottom; (2) partial clonus (PC): clonic seizure in face, head or forelimbs; (3) generalized clonus (GC): sudden loss of upright posture, whole-body clonus including all limbs and tail, rearing and autonomic signs; and (4) tonic-clonic seizure (TC): generalized seizure up to tonic hind-limb extension and death. Latencies to (2)–(4) are used to calculate individual seizure scores (ISS), where factors weight relative severity: ISS=1000/(0.2 × PC-latency+0.3 × GC-latency+0.5 × TC-latency).^55, 56, 57^

### Statistical methods

Case-control analysis and test for deviation from Hardy–Weinberg equilibrium was performed using PLINK1.07.^58^ Statistics for human phenotype–genotype associations and mouse studies were conducted with SPSS v.17.0 (IBM-Deutschland, Munich, Germany), STATA MP-v.13.1 (StataCorp, College Station, TX, USA) and Prism4 (GraphPad-Software, San Diego, CA, USA). Statistical tests used are always given in figure legends. Data are presented as mean±s.e.m., statistical significance was set to *P*=0.05.

## Results

### A normal *AMBRA1* genotype, rs3802890-AA, is associated with autistic traits predominantly in female schizophrenic individuals

Only one directly genotyped and—according to our PGAS SOP^23^—suitable SNP, *AMBRA1*-rs3802890-A/G, was available in our array. Case–control analysis (1105 schizophrenic versus 1258 healthy GRAS subjects) yielded comparable genotypic and allelic chi-square comparison (MAF=0.31; controls: AA=607, AG=532, GG=119; cases: AA=507, AG=505, GG=93; genotypic: *χ*^2^=2.974, df=2, *P*=0.226; allelic: *χ*^2^=0.242, df=1, *P*=0.623). Thus, rs3802890 is not associated with the schizophrenia diagnosis.

Next, PGAS was performed with rs3802890-AA/-GG and PAUSS^24^ as quantitative measure of autistic traits (Figure 1a). In previous work, we have demonstrated that autistic features cross diagnostic borders and can be quantified not only in ASD, but also in schizophrenia and other diseases as well as in healthy individuals.^21, 23, 24^ For quantification, we developed the PAUSS, a dimensional instrument based on PANSS,^42^ capturing the continuous nature of autistic behaviors.^24^ PGAS revealed an association: AA carriers display higher PAUSS scores than GG subjects (*P*=0.039). Interestingly, when separating genders, the PAUSS association remains significant only for females and, likewise, most PAUSS sub-items show this trend in females but not males (Figures 1b and c).

### A role of *AMBRA1*-rs3802890-AA for female autistic features is confirmed in a general population sample

Even though this highly targeted approach to an association of only one available *AMBRA1* SNP with autistic traits in schizophrenic individuals was already encouraging, we aimed at replication of this finding in a general population sample. For this, a social support score, derived from ISI,^43^ was used as proxy phenotype, expected to be low in individuals with autistic features.^23^ Cross-validation of social support (operationalized as the self-reported number of individuals a person can rely on in case of emergency) with PAUSS in the discovery sample (GRAS) yielded a high negative Spearman rank correlation (Figure 1d), underlining the relevance of this proxy for autistic features. Again, the social support score disclosed a genotype effect (rs3802890-AA/-GG) in both discovery and replication sample, more pronounced in females (Figures 1e and f). Thus, in two independent human cohorts, a single normal variant in the *AMBRA1* gene, rs3802890, is associated with autism-related behaviors predominantly in females.

### Consequence of *AMBRA1*-rs3802890-A versus G on mRNA expression in human PBMC and *in silico* prediction of potential underlying mechanisms

In some subjects, PBMCs were available for *AMBRA1* mRNA analysis. Although female GG (*N*=14) versus male GG (*N*=33) carriers had higher expression levels (0.0059 versus 0.0045 *AMBRA1/GAPDH*; *P*=0.05), AA carriers of both genders (males *N*=35; females *N*=33) did not differ in their level (males 0.0049; females 0.0046; *P*=0.62), which was comparable to that of male GG carriers. Comparing both genders, *AMBRA1* mRNA expression in PBMC of AA (risk SNP) relative to GG carriers is reduced in females but not males (*P*=0.017; Figure 1g), possibly indicating partial loss-of-function of *AMBRA1* in AA females. This relative reduction found in PBMC of women resembles the situation in cortex of female mice: normal WT females have higher Ambra1 expression than WT males, whereas *Ambra1*^+/−^ females show stronger relative Ambra1 reduction compared to *Ambra1*^+/−^ males.^29^

**Figure 2:**
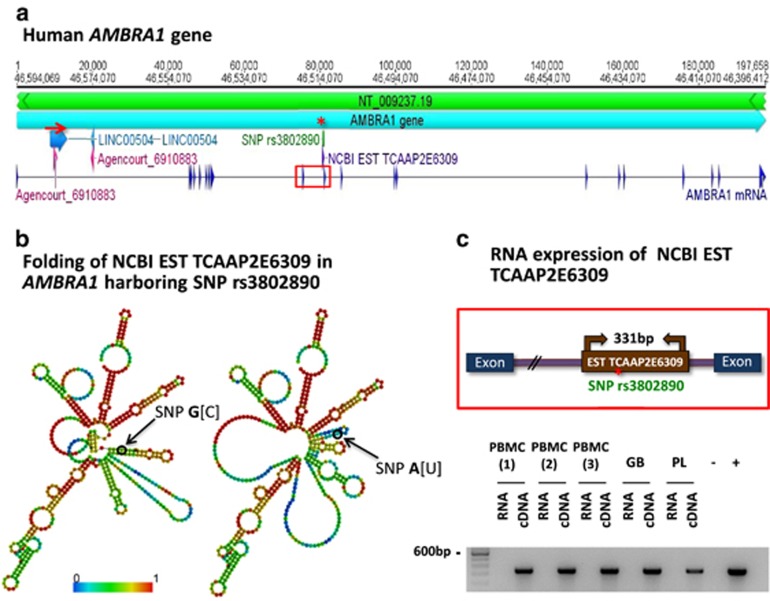
*AMBRA1-*rs3802890 G/A: *in silico* approach to mechanistic insight. (**a**) Detailed map showing the human *AMBRA1* gene (NCBI-accession: 55626; chromosomal location: Chr.11: 46 396 412–46 594 069). The *AMBRA1* mRNA (21 exons) is mapped at the bottom. The location of *AMBRA1*-rs3802890 is indicated by a red asterisk. The region shared between all chromosomes (nucleotides from around 9530 to 11 030 on *AMBRA1*; red arrow) is indicated. Similar to man, the murine *Ambra1* gene region matches on all chromosomes (nucleotides from around 68 110–69 940 on murine *Ambra1*). However, the chromosomal match region of *AMBRA1/Ambra1* is different between both species. Both match regions show similarities to expressed sequence tags (EST) from NCBI database (best match EST gi|22688027 in human and gi|44663783 in mice; both with full-length alignment) and to specific lncRNAs in Refseq database (best match human: Refseq Accession NR_126435.1 named LINC00504; best match in mice: Refseq Accession NR_131899.1 named Mrqpra6). (**b**) RNA folding of human *AMBRA1* transcribed EST comparing the G allele with the A allele. The presentation of thermodynamic ensemble folding stresses differences in the obtained structure. The color code indicates pairing propensities. The fold is further supported by reoccurring similar differences comparing several foldings and also lengths using software mFold. As template for folding, the full RNA sequence of EST from myelogenous leukemia cells (496 nucleotides long, 98% identity; full-length alignment; genbank accession BM149074.1; pediatric acute myelogenous leukemia cell (FAB M1) EST TCAAP2E6309) is shown. This RNA encodes no protein, has no introns/exons and has no complementary match in any other chromosomal region. (**c**) EST TCAAP2E6309 expression in all tested tissues: RNA was isolated from peripheral blood mononuclear cells (PBMC), human glioblastoma tissue (GB), and human placenta (PL). PCR was performed from cDNA. To exclude false positive results by genomic DNA contamination, several controls were performed (DNase digestion; respective RNA amplification). Genomic DNA from whole blood was used as positive control (+) and ddH_2_O as negative control (−).

A detailed map of human *AMBRA1* gene is explained in Figure 2a. Exploring the location of the intronic rs3802890, a similarity to expressed sequence tags (EST) from NCBI database arises (Figure 2a). The predicted RNA folding of the transcribed EST TCAAP2E6309, covering the SNP region, is remarkably influenced by the presence in rs3802890 of G versus A (Figure 2b). As we find EST TCAAP2E6309 RNA expressed in PBMC and other human tissues (Figure 2c), relevance of this lncRNA for *AMBRA1* mRNA or protein levels may be assumed.

Together, these data suggest that *AMBRA1* likely shapes autistic behavior also in humans in a sexually dimorphic way. This across-species unique female autism generation by a defined genetic trait that appears to cause partial loss-of-function encouraged us to continue searching for further autism-specific readouts in our *Ambra1*^+/−^ mouse model.

### Pheromone-based social preference is reduced in *Ambra1*^*+/−*^ females

**Figure 3:**
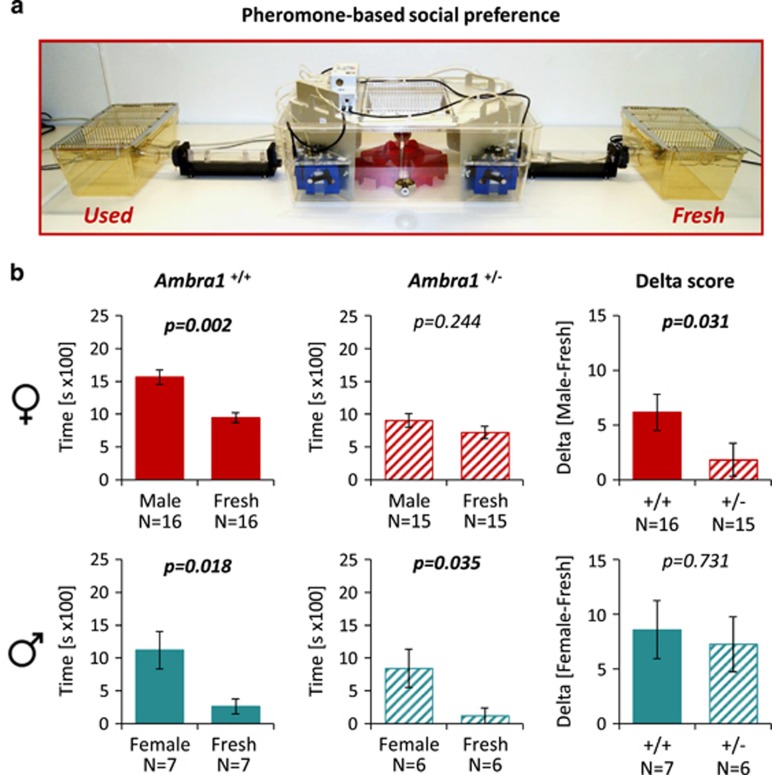
Impaired pheromone preference in female *Ambra1* mutants. (**a**) Intellicage apparatus with connected social boxes. (**b**) Time spent in social boxes with used or fresh bedding or delta difference scores for the indicated genotypes. Upper row females; lower row males; mean±s.e.m. presented. Within-group comparisons performed with paired *t*-tests, between-group with Mann–Whitney *U*-tests; all tests two-tailed.

Sex pheromones have an important role in social behavior throughout the animal kingdom.^59, 60, 61, 62^ Mice typically favor a social context that contains pheromones of the opposite gender. In autistic phenotypes, social interest, approach and communication as well as understanding of social signals are compromised.^1, 2, 3, 4, 62^ We therefore designed a novel intellicage set-up to test pheromone preference as potential autism-relevant readout in *Ambra1*^+/−^ mice. Upon free choice between a social box containing used bedding from mice of the opposite gender and another social box with fresh bedding only, WT male and female and *Ambra1*^+/−^ male mice behave as expected, namely choose to stay longer in the respective ‘pheromone box’. In contrast, *Ambra1*^+/−^ females fail to show this preference (Figure 3).

### MRI analysis reveals brain enlargement in *Ambra1*^*+/−*^ mice

**Figure 4:**
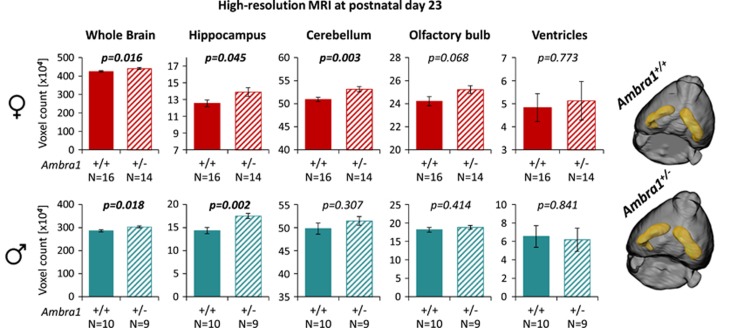
Brain enlargement in prepubertal *Ambra1* mutants of both genders. Shown are results of high-resolution magnetic resonance imaging (MRI) (T2-weighted). Brain regions of interest (whole brain, hippocampus, cerebellum, olfactory bulb, ventricles) in 23day-old female (upper row) and male (lower row) mice of both genotypes are presented; mean±s.e.m.; two-tailed unpaired *t*-tests. Right side: Representative pictures of 3D-reconstructed brains of both genotypes illustrate brain enlargement in mutants.

**Figure 5:**
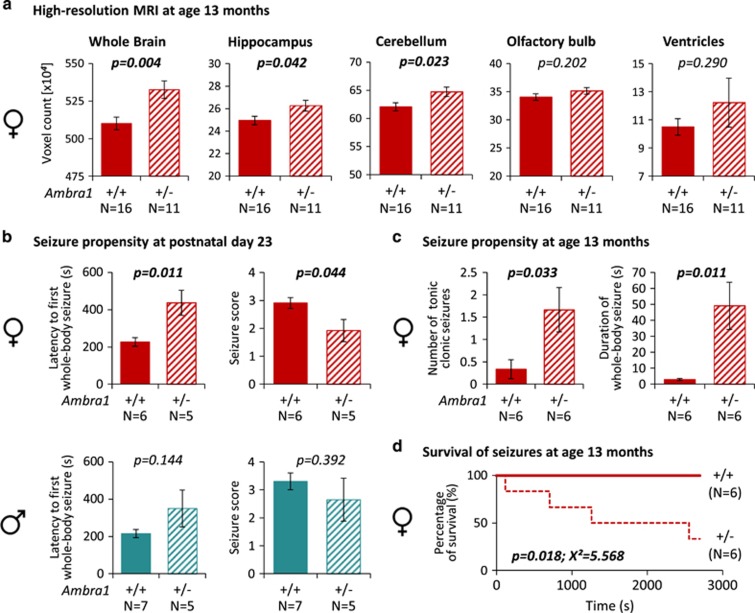
Persistent brain enlargement and altered pentylentetrazol (PTZ)-induced seizure propensity in female *Ambra1* mutants. (**a**) Brain regions of interest (whole brain, hippocampus, cerebellum, olfactory bulb, ventricles) were analyzed by high-resolution magnetic resonance imaging (MRI) in 13-months-old female *Ambra1*^+/−^ and WT mice; mean±s.e.m.; two-tailed unpaired *t*-tests. (**b**) PTZ-induced seizure propensity (intraperitoneal injection of 50 mg kg^−1^) in 23-day-old WT and *Ambra1*^+/−^ mice of both genders; mean±s.e.m. presented; two-tailed unpaired *t*-tests. (**c**) PTZ-induced seizure propensity (intraperitoneal injection of 50 mg kg^−1^) in 13-months-old female WT and *Ambra1*^+/−^ mice; mean±s.e.m. presented; two-tailed unpaired *t*-tests. (**d**) Survival of PTZ-induced seizures (intraperitoneal injection of 50 mg kg^−1^) in 13-months-old female WT and *Ambra1*^+/−^ mice; Kaplan–Meier survival analysis.

Brain enlargement has been described both in children and adults with ASD.^5, 6, 7, 8^ Recently, brain volume overgrowth in children was linked to the emergence and severity of autistic social deficits.^7^ We measured by high-resolution MRI (T2-weighted) brain dimensions in *Ambra1*^+/−^ versus WT mice. Whole brain and hippocampus were enlarged in male and female mutants at postnatal day 23 (around puberty). Cerebellum was increased in female *Ambra1*^+/−^ mice only (Figure 4). Sizes of olfactory bulb and ventricles in *Ambra1*^+/−^ mice were similar to WT. Repeated examination of females at age 13 months revealed persistence of the increased brain dimensions (Figure 5a). We note that *Ambra1*^+/−^ mice are the first autism model showing autism-typical brain enlargement. Regarding this particular readout, genders were comparable, uncoupling in this model autism-like behavior (only females) from brain dimensions.

### Female *Ambra1*^*+/−*^ mice show altered seizure propensity

Another frequently observed trait, connected with autistic behaviors, not only in syndromic forms of autism, is epileptic seizures.^9, 10^ Most likely, seizure predisposition reflects the autism-pathognomonic neuronal excitation–inhibition dysbalance.^4, 11, 12, 13, 14, 15^ In our *Ambra1*^+/−^ model, prepubertal female mutants displayed reduced response to PTZ, namely longer latency to the first whole-body seizure and decreased seizure score compared to female WT (Figure 5b). This early resistance turned into the opposite response at older age: Number of tonic-clonic seizures and duration of whole-body seizures were enhanced in 3-months (data not shown) and 13-months-old *Ambra1*^+/−^ females versus WT, also resulting in reduced survival (Figures 5c and d). Male mutants did not differ from WT at any time point investigated. Together, these data support *Ambra1*^+/−^mice as multidimensional model of human autism.

## Discussion

We previously reported in female *Ambra1*^*+/−*^ mice a discrete behavioral trait, reminiscent of human ASD.^29^ In the present study, we extend this finding, showing for we believe the first time that *AMBRA1* may—in likewise sexually dimorphic manner—be relevant also in humans for the expression of a female autistic phenotype. The female preponderance, unique thus far in autism genetics, may even help illuminating some general molecular underpinnings of gender susceptibility to brain disease. Remarkably, in a highly targeted association approach, using the only *AMBRA1* SNP available for analysis, rs3802890-A/G, we find in two independent populations, the GRAS sample of schizophrenic individuals and the SHIP sample of general population subjects, relevance for this marker regarding autistic traits in women. Partial loss-of-function, reflected by a relative decrease in *AMBRA1* mRNA levels in PBMC of female risk genotype (AA) carriers, may suggest an underlying autism-causing mechanism similar to that in heterozygous mice where Ambra1 reduction was stronger in female than male mutant cortex.^29^

The question of how AMBRA1/Ambra1 reduction may influence synaptic function, thereby causing the autism-pathognomonic neuronal excitation–inhibition dysbalance,^4, 11, 12, 13, 14, 15^ still remains unanswered. We may, however, speculate that reduced autophagy at synaptic terminals,^30, 31, 63^ likely more pronounced in *Ambra1*^+/−^ females,^29^ influences synaptic protein turnover and function in a gender-specific manner. In fact, females may be particularly sensitive to reduced autophagy as suggested also by a recent paper reporting higher basal autophagy activity in the brain of neonatal female as compared to male rats.^64^ In this sense, AMBRA1/Ambra1 adds to the number of proteins shown to underlie sexually dimorphic effects on the brain.^65, 66^

In a first *in silico* search for mechanisms, we saw that the lncRNA, covering the SNP region, shows highly diverse folding upon presence of G versus A allele. This pronounced structural effect may influence *AMBRA1* mRNA and/or protein stability and will be subject for further investigation.

Returning to the *Ambra1*^+/−^ mouse model, we extend our earlier findings^29^ to crucial, additional autistic features, so far not systematically addressed in genetic models of autism, namely early brain enlargement, altered propensity towards epileptic seizures and reduced pheromone preference.

Brain enlargement is a consistently reported feature in human autism, both in adults and children.^5, 6, 7, 8^ Already upon first description of autism, increased size of the head was observed in affected children.^67^ The substrate underlying the enlarged brain has remained obscure, and the *Ambra1*^+/−^ mouse model may now help to approach this question. Interestingly, we found *Ambra1*^+/−^-associated brain enlargement in both genders, thus uncoupled from the predominantly female behavioral phenotype. This finding may be important in connection with recent suggestions, based on genetically undefined autistic children, where brain volume overgrowth was linked to the emergence and severity of autistic social deficits, predicting a later autism diagnosis based on MRI deep-learning algorithms.^7^ This obvious discrepancy should stimulate further investigations considering gender, genetics and biological ASD subgroups.

Epileptic seizures are frequently observed, not only in syndromic autism, where they are often intractable,^9, 10^ and mirror neuronal excitation/inhibition dysbalance.^4, 11, 12, 13, 14, 15^ Also here, *Ambra1*^+/−^ mice revealed a striking sexual dimorphism and may serve as future model to study and treat autism-associated epilepsy.

Olfactory deficits in human autism have been reported, even though the literature is scarce, heterogeneous and inconsistent, likely reflecting subject selection and assessment biases or other methodological limitations, including statistical power issues.^68^ We note, however, that compared to humans, pheromone preference in mice represents a more prominent component of their social behavioral repertoire, thus more vulnerable to be disturbed in autism-like phenotypes of this species.

To conclude, our data suggest a fascinating sexual dimorphism regarding the role of the autosomal *AMBRA1/Ambra1* gene for autistic traits across species. In humans, it will for instance be interesting to systematically screen ASD patients for *AMBRA1* mutations, particularly female autists. *Ambra1*^+/−^ mice may serve as a novel multilayered construct-valid genetic model of human autistic phenotypes.
